# Potential Relationships between the Median Nerve Cross-Sectional Area and Physical Characteristics in Unilateral Symptomatic Carpal Tunnel Syndrome Patients

**DOI:** 10.3390/jcm12072515

**Published:** 2023-03-27

**Authors:** Akira Ikumi, Yuichi Yoshii, Takamasa Kudo, Sho Kohyama, Takeshi Ogawa, Yuki Hara, Tomoo Ishii

**Affiliations:** 1 Department of Orthopaedic Surgery, Tsukuba University Hospital, Tsukuba 305-8576, Japan; 2 Department of Orthopaedic Surgery, Tokyo Medical University Ibaraki Medical Center, Ami 300-0395, Japan; 3 Department of Orthopaedic Surgery, Kikkoman General Hospital, Noda 278-0005, Japan; 4 Department of Orthopaedic Surgery, Mito Medical Center Hospital, Ibaraki 311-3193, Japan; 5 Department of Orthopaedic Surgery, National Center of Neurology and Psychiatry, Kodaira 187-8551, Japan

**Keywords:** carpal tunnel syndrome, cross-sectional area, physical characteristics, ultrasonography, unilateral symptom

## Abstract

Background: The present study investigated the relationships between the median nerve cross-sectional area (CSA) and physical characteristics in patients with unilateral symptomatic carpal tunnel syndrome (CTS). Methods: Height, weight, body mass index (BMI), disease duration, results of electrodiagnostic testing (EDX), and median nerve CSA at the level of the wrist crease were recorded in 81 patients with CTS who presented with symptoms on only one side. Correlation coefficients between median nerve CSA and physical characteristics, disease duration, and results of EDX were analyzed. Results: Median nerve CSA at the wrist crease (mm^2^) was significantly larger on the symptomatic side (14.1 ± 3.8) than on the asymptomatic side (11.5 ± 2.9). Median nerve CSA correlated with body weight (correlation coefficient = 0.39) and BMI (correlation coefficient = 0.44) on the asymptomatic side, but not on the symptomatic side. These correlations were slightly stronger in females (correlation coefficient = 0.46) than in males (correlation coefficient = 0.40). No correlations between median nerve CSA and disease duration and the results of EDX were observed in both sides. Conclusions: In patients with unilateral symptomatic CTS, median nerve CSA correlated with BMI only on the asymptomatic side. The present results suggest that the relationship between median nerve CSA and BMI in CTS is significant until symptom onset but may be masked by edema and pseudoneuroma after its onset. A higher BMI is associated with a larger CSA of the median nerve, which may be a risk factor for the development of CTS.

## 1. Introduction

Carpal tunnel syndrome (CTS) is caused by compression of the median nerve within the carpal tunnel (CT) and is the most common entrapment neuropathy [1]. The general incidence of CTS is estimated to be 0.2–4% [1,2,3,4]. Clinical symptoms, a physical examination, electrodiagnostic testing (EDX), and ultrasonography (US) are commonly combined to diagnose CTS because of the lack of a consensus on a single diagnostic modality [5]. US was previously reported to be as accurate as other reference standards, such as the 6-item CTS symptoms scale (CTS-6) and EDX, with 91% sensitivity and 94% specificity [6,7,8,9]. The median nerve cross-sectional area (CSA) at the CT is the most commonly used ultrasonographic parameter for the diagnosis and severity evaluation of CTS [10,11,12]. In CTS, nerve compression results in a localized circulatory disturbance with the collapse of the blood–nerve barrier increasing endoneurial fluid pressure, with resultant nerve swelling and further impairments in local blood flow [13].

CTS has a bimodal distribution and is more common in women than in men, with onsets after the ages of 40 and 70 years, respectively [14]. Although most cases of CTS are idiopathic, it sometimes occurs secondary to a lesion in the CT, such as a ganglion, tenosynovitis due to rheumatoid arthritis, dialysis amyloidosis, or pyogenic tendonitis. Several risk factors of CTS such as diabetes mellitus, menopause, hypothyroidism, obesity, arthritis, and pregnancy had been mentioned in previous research. Shiri et al. described the relationships between hypothyroidism and CTS [15]. Padua et al. clearly does support the relevance of pregnancy as a risk factor [16]. Pourmemari et al. suggested that both Type 1 and Type 2 diabetes mellitus are risk factors for CTS [17]. Shiri et al. calculated that being overweight increased the risk of carpal tunnel decompression by a factor of 1.5 [18]. Although various risk factors of CTS have been examined in the past, the factors contributing to symptom onset in CTS remain unclear.

CT release is usually effective for CTS patient whose symptoms do not improve with conservative therapy, but symptoms are not relieved to a satisfactory level in about 25% of cases [19]. The recurrence rate after CT release is also reported in 57% about 2 years later [20]. To explain the surgical failure, several pathogeneses of CTS have been proposed in the past. Festen-Schrier and Amadio described the changes in subsynovial connective tissue (SSCT) [21]. Vilensky et al. described the changes in vascular morphology [22]. Sunderland described the increasing intra-computed tomography pressure [23]. Jinrok et al. described the changes in the nerves serving the epineurium and the median nerve itself (neuropathy) [24]. Stecco et al. described that unbalanced tension of epimysial fasciae can affect the paraneural sheath, limiting nerve displacement, and consequently this must be included in carpal tunnel syndrome pathogenesis [25]. The question of whether there is a relationship between physical characteristics and median nerve size in considering mechanical factors such as SSCT remains unclear.

We previously examined the clinical relevance of US and EDX findings of the median nerve in unilateral symptomatic CTS patients. Median nerve CSA at the wrist crease level on US was significantly larger on the symptomatic side; the area under the curve value was 0.74, and the cut-off value was 11.9 mm^2^ [26]. Few studies have compared the asymptomatic and symptomatic sides in the same CTS patients. We hypothesized that physical characteristics, such as body mass index (BMI), contribute to the development of CTS symptoms. Therefore, the present study investigated the relationships between median nerve CSA on US and physical characteristics in unilateral symptomatic CTS patients and the incidence of risk factors for CTS.

## 2. Materials and Methods

The protocol for the present study was reviewed and approved by our Institutional Review Board (approval number T2020-0061). Informed consent was obtained from all patients for inclusion in the present study.

The bilateral wrist joints of 81 unilateral symptomatic idiopathic CTS patients (162 wrists, 26 males, 55 females, 30–89 years, mean 65.9 years) were evaluated. Patients suspected of having secondary CTS due to chronic kidney disease, thyroid disease, diabetes mellitus, and rheumatoid arthritis were excluded from the analysis. Patients with a history of upper limb surgery were also excluded.

CTS was diagnosed based on clinical symptoms and the findings of EDX and US as previously described [26,27]. We defined unilateral symptomatic CTS as patients with characteristic symptoms on one side of the hand and no symptoms on the other side. A single experienced hand surgeon (YY) discriminated between the asymptomatic and symptomatic sides based on an interview and clinical findings. Each patient’s physical characteristics, namely, height, body weight, and BMI, disease duration, and the results of EDX (the latencies of the compound muscle action potential (CMAP) and the sensory nerve action potential (SNAP) were retrospectively recorded from medical records.

The EDX was performed using a standard electromyography system (Neuropack MEB-2208, Nihon Kohden Co., Tokyo, Japan). All studies were performed by a clinical technician who was blinded to clinical symptoms. At the time of the EDX, room temperature was maintained at 27 °C. Among patients with cold hands, the hands were warmed to bring the skin temperature closer to room temperature. In the motor conduction study, the CMAP of the abductor pollicis brevis muscle was recorded. CMAP was induced by a stimulation 7-cm proximal to the recording electrode. In the sensory conduction study, a stimulating electrode was placed at the index finger, and a recording electrode was placed 14-cm proximal to the stimulating electrode. The SNAP was recorded. The latencies of CMAP and SNAP were measured. The result of distal latency of CMAP (ms) was divided into six categories, and the correlation was analyzed using Spearman’s rank correlation coefficient as follows: under 4.0 = 1, 4.1 to 6.0 = 2, 6.1 to 8.0 = 3, 8.1 to 10.0 = 4, over 10.1 = 5, unmeasurable = 6. The result of sensory nerve conduction velocity (m/s) was divided into four categories and the correlation was analyzed using Spearman’s rank correlation coefficient as follows: over 40 = 1, 20 to 39.9 = 2, 0 to 19.9 = 3, unmeasurable = 4.

**Figure 1:**
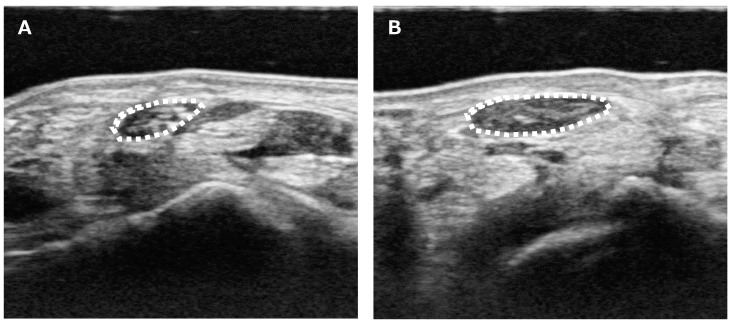
Ultrasound measurement of the median nerve CSA. (**A**) Unaffected side. (**B**) Affected side. CSA was measured as the only parameter for the image analysis. Median nerve CSA was measured at the wrist crease level. The median nerve was outlined (white dotted line), and its area was calculated.

Median nerve CSA was measured using the following method: US was routinely performed on patients with suspected CTS to differentiate abnormalities around the CT. During the diagnostic process, median nerve CSA was measured at the wrist crease level (Figure 1). Each patient was asked to sit and place their forearm on the table with the palmar side up. An ultrasound scanner (Hi Vision Avius; Hitachi Aloka Medical, Ltd., Tokyo, Japan) equipped with a linear array transducer was set to a depth of 20 mm. The frequency of the transducer was 15 MHz. Cross-sectional ultrasonographic images of the CS were analyzed using ImageJ Software (National Institutes of Health, Bethesda, MD, USA). The median nerve was outlined, and its area was calculated. US was performed by a single hand surgeon (YY) who is certified as a specialist and instructor by the Japanese Society of Surgery of the Hand.

Statistical analyses were performed using the methods described herein. Results are expressed as the mean ± standard deviation. Differences between the symptomatic and asymptomatic sides and between the sexes were evaluated with Welch’s *t*-test. Values of *p* < 0.05 were considered to be significant. Pearson’s product–ratio correlation analysis was used to measure correlation coefficients between median nerve CSA and height, weight, BMI, and disease duration on the symptomatic and asymptomatic sides. Spearman’s rank correlation coefficient was used to measure correlation coefficients between median nerve CSA and the results of NCS on the symptomatic and asymptomatic sides. According to Cohen’s recommendation, an absolute value of correlation coefficient of 0.1 is classified as small, an absolute value of 0.3 is classified as medium, and one of 0.5 is classified as large [28]. All analyses were performed using Bellcurve for Excel (version 2.14) and IBM SPSS statistics (version 28.0.1.1).

## 3. Results

Table 1 shows patient demographics. Height and body weight were significantly larger in males than in females (*p* < 0.01). No significant differences were observed in age or BMI by sex (*p* = 0.43 in age, and *p* = 0.75 in BMI).

The average disease duration was 17.0 ± 27.9 (range 1 to 120) months.

Table 2 shows the results of EDX categorization. There were significant differences between the symptomatic and asymptomatic side (*p* < 0.01). Both distal latency and sensory nerve conduction velocity were significantly worth in symptomatic side.

**Figure 2:**
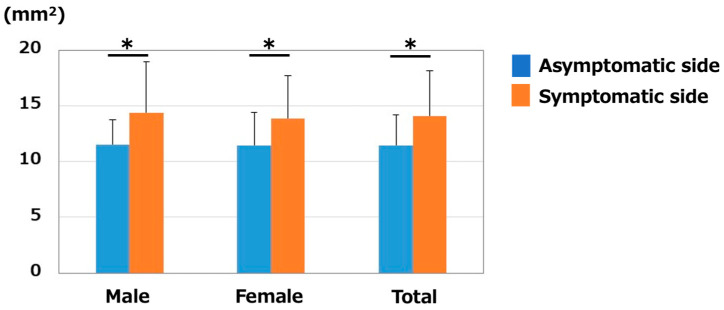
Median nerve CSA (mm^2^). CSA was significantly larger on the symptomatic side in both males and females. * *p* < 0.05 between the sides.

The median nerve CSA of the asymptomatic/symptomatic sides (mm^2^) were 11.2 ± 2.6/14.0 ± 4.4 in males, 11.6 ± 3.0/14.1 ± 3.6 in females, and 11.5 ± 2.9/14.1 ± 3.8 in total. Median nerve CSA was significantly larger on the symptomatic side than on the asymptomatic side in all comparisons (male, female, and total, *p* < 0.01) (Figure 2). No significant differences were noted between the sexes (*p* = 0.58 on the asymptomatic side, and *p* = 0.93 on the symptomatic side).

**Figure 3:**
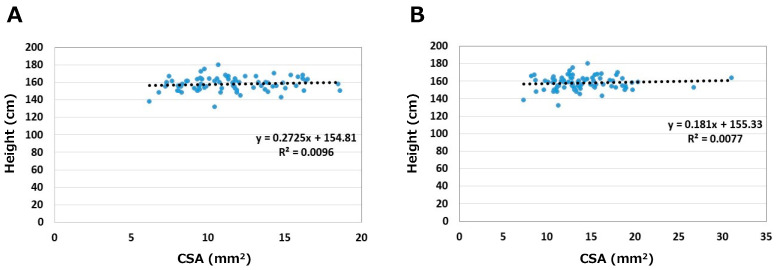
Relationships between height and CSA. (**A**) Asymptomatic side. (**B**) Symptomatic side.

The relationships between height and CSA are shown in Figure 3. Correlation coefficients were 0.10 on the asymptomatic side and 0.09 on the symptomatic side. Height did not correlate with CSA on either side.

**Figure 4:**
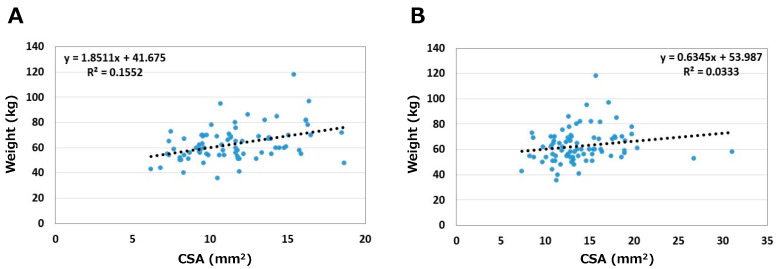
Relationships between weight and CSA. (**A**) Asymptomatic side. (**B**) Symptomatic side.

The relationships between body weight and CSA are shown in Figure 4. Correlation coefficients were 0.39 on the asymptomatic side and 0.18 on the symptomatic side. Weight medium correlated with CSA on the asymptomatic side only (*p* < 0.05).

**Figure 5:**
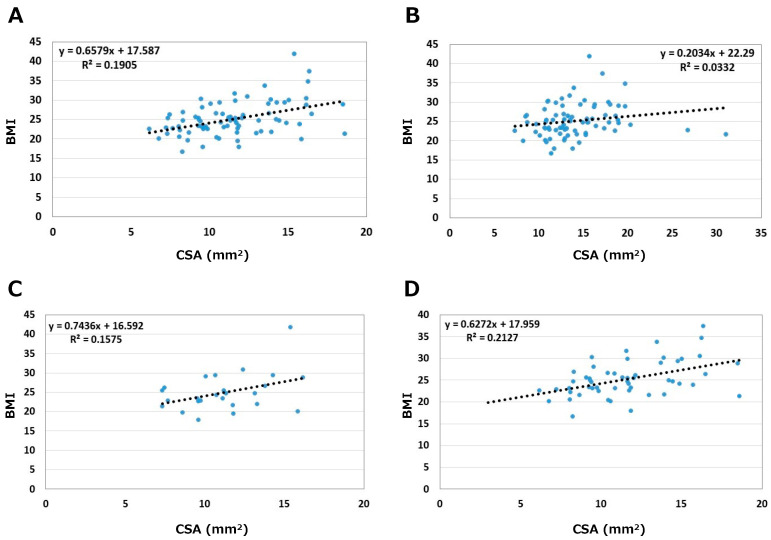
Relationships between BMI and CSA. (**A**) Asymptomatic side. (**B**) Symptomatic side. (**C**) Male. (**D**) Female.

The relationships between BMI and CSA are shown in Figure 5. Correlation coefficients were 0.44 on the asymptomatic side and 0.18 on the symptomatic side. On the asymptomatic side, correlation coefficients between BMI and CSA by sex were 0.40 in males and 0.46 in females. BMI medium correlated with CSA on the asymptomatic side in both sexes (*p* < 0.05); slightly stronger in females than in males.

Correlation coefficients between median nerve CSA and disease duration were −0.14 on the asymptomatic side and −0.08 on the symptomatic side. There was no correlation between median nerve CSA and disease duration on both sides (*p* = 0.24 on the asymptomatic side and *p* = 0.51 on the symptomatic side). 

Correlation coefficients between median nerve CSA and distal latency of the EDX were 0.13 on the asymptomatic side and 0.08 on the symptomatic side. Correlation coefficients between median nerve CSA and sensory nerve conduction velocity of the EDX were 0.19 on the asymptomatic side and 0.15 on the symptomatic side. No correlations were observed between median nerve CSA and the results of EDX on both side (*p* > 0.05). 

## 4. Discussion

In the present study, median nerve CSA was significantly larger on the symptomatic side. Furthermore, weight/BMI correlated with median nerve CSA on the asymptomatic side. On the other hand, physical characteristics did not correlate with median nerve CSA on the symptomatic side. No significant differences were observed in CSA between the sexes even though physical characteristics significantly differed.

US on median nerve CSA have been compared between healthy controls and patients with peripheral neuropathy. A systematic review reported a mean normative reference value of 8.74 mm^2^ for median nerve CSA at the CT entrance, which is approximately the same site as the wrist crease assessed in the present study (n = 1863) [29]. Furthermore, the systematic review found no correlation between age or sex and reported a value for median nerve CSA at the CT inlet that appeared to be similar between each region. On the other hand, individual and site-specific differences in median nerve CSA were not small. Among studies measuring normal median nerve CSA at the CT inlet level, individual values widely ranged between 7 and 10 mm^2^ (lower and upper bounds of 95% CI, respectively) [29]. Nakamichi et al. measured the median nerve CSA of CTS patients at three different levels (the distal edge of the flexor retinaculum, the hook of the hamate, and at the wrist crease) and suggested that the median nerve had an hourglass-like shape [30]. Since there are large individual differences in normal values and CSA values vary depending on the measurement site, this study was validated by the difference in median nerve CSA between the symptomatic and asymptomatic sides in unilateral symptomatic CTS patients. Although median nerve CSA at the CT inlet significantly differed between the asymptomatic and symptomatic sides in patients with unilateral symptomatic CTS, CSA on the asymptomatic side (11.5 ± 2.9 mm^2^) was also higher than normal values in previous studies. Different CSA measuring methods in each study may have affected the data obtained; however, CTS often occurs bilaterally, and the asymptomatic side may have also been affected by constriction at the CT. The usefulness of CSA measurements in patients with unilateral symptomatic CTS is that interventions at the time of symptom onset on one side may prevent the development of symptoms on the asymptomatic side. To validate the usefulness of median nerve CSA in unilateral symptomatic CTS patients in the future, it is necessary to define whether the measured median nerve CSA is an enlarged condition for each individual because of individual differences in median nerve CSA. Hobson-Webb et al. proposed a method to quantify local nerve enlargement by comparing the wrist and forearm CSA of the median nerve and reported that the wrist-to-forearm CSA ratio improved the diagnostic accuracy of CTS over a single CSA measurement at the CT inlet [31]. Therefore, additional studies are needed to validate the relationship between median nerve CSA and the development of CTS by using CSA ratios at multiple sites in the same patient and also by using the CSA ratio between the asymptomatic and symptomatic sides.

The cut-off value for median nerve CSA on US to diagnose CTS reportedly varied between 9 and 14 mm^2^, with sensitivity of 57–94% and specificity of 57–98% [9,32,33,34]. Furthermore, a meta-analysis reported pooled sensitivity and specificity of US for the diagnosis of CTS of 77.6% (95% CI 71.6–83.6%) and 86.8% (95% CI 78.9–94.8%), respectively. Another recent meta-analysis revealed that its diagnostic odds ratio may be as high as 31.11 (95% CI, 20.42–47.40) [35,36]. Although sensitivity and specificity widely varied among these studies, the main difficulty associated with diagnosing CTS by median nerve CSA is that it does not take into account differences in normative nerve sizes due to race, sex, and body characteristics. Although there were significant sex differences in physical characteristics in the present study, median nerve CSA did not significantly differ between males and females on the asymptomatic and symptomatic sides. Furthermore, based on the lack of a correlation between height and CSA, median nerve CSA may not correlate with body size, as represented by height. On the other hand, since the CT area depends on the morphology and size of the carpal bones that comprise the CT, the area ratio of the median nerve to the CT may be larger in females than in males. This may be one reason why CTS occurs more frequently in females.

Regarding the relationships between physical characteristics and median nerve CSA, previous studies suggested a positive correlation between BMI and median nerve CSA [37,38,39,40,41]. Cartwright et al. reported that mean age based on BMI was a major parameter for predicting median nerve CSA in healthy subjects [42]. Other studies demonstrated that BMI and diabetes were associated with larger median nerves at the CT [39,40,41]. An MRI-based median nerve CSA study also showed that CSA increased with BMI in non-CTS patients, and obese patients with CTS had larger CSA values at the CT inlet level [42]. The mechanism by which obesity/high BMI increases median nerve CSA is unknown. High lipid conditions due to obesity may be involved. Intraneural adipose cells are present inside nerves [43]. Reina et al. examined the distribution of intraneural adipose cells in the sciatic nerve using a scanning electron microscope and reported that adipose tissue inside a nerve surrounded the fascicles to form adipose sheaths that separated the fascicles from one another [44]. High BMI due to obesity promotes the proliferation of adipocytes, including intraneural adipose cells. An increase in the median nerve CSA with obesity may result in median nerve constriction and the development of CTS. CTS, which occurs via the same mechanism as the above condition, is also observed in patients with lipofibromatous hamartoma (LFH) of the median nerve. LFH is a condition in which fat is deposited in the nerves, and the pathogenesis of this disease is considered to be congenital and slowly progressive [45]. Although previous studies reported that higher BMI and obesity were associated with a higher incidence of CTS [46,47,48], BMI/weight did not correlate with median nerve CSA on the symptomatic side. The prolonged constriction of peripheral nerves results in the formation of a pseudoneuroma (enlargement of median nerve CSA) proximal to the constriction site. The formation of a pseudoneuroma may mask the relationship between median nerve CSA on the symptomatic side and BMI. To verify this hypothesis, median nerve CSA changes on the asymptomatic side need to be tracked in future studies.

There are several limitations that need to be addressed. First, our measurements were only taken at the proximal CT level. To verify the correlations between median nerve CSA and physical characteristics on the symptomatic side, CSA may need to be measured at multiple sites, such as the CT outlet or forearm. Second, the sample size of this study was insufficient in the extent to which physical characteristics affect median nerve CSA compared with other variables which might have an impact on the CSA measurements such as patient age or any independent measure of the severity or duration of CTS. To verify whether physical characteristics truly affect median nerve CSA, additional study by accumulating cases to conduct multivariate analysis is necessary in the future. Thirdly, since many patients with CTS exhibit symptoms in both wrists, the unaffected side in the present study may develop CTS in the future. However, the strength of the present study is that we compared pre-onset and post-onset conditions. Another limitation is that patient backgrounds between sexes were not unified by numbers or physical characteristics. More accurate validation is needed to accumulate more cases in the future. Finally, there is the potential risk of a measurement bias because the CSA measurement by US was performed by one evaluator. Although the diagnosis was confirmed by an electrophysiological test, blind ultrasonography may need to be considered in future studies.

## 5. Conclusions

BMI/weight medium correlated with median nerve CSA on the asymptomatic side in unilateral symptomatic idiopathic CTS patients. The present results suggest that the relationship between median nerve CSA and BMI in CTS is significant until symptom onset but may be masked by edema and pseudoneuroma after its onset. A higher BMI is associated with a greater CSA of the median nerve, which may be a risk factor for the development of CTS.

## Figures and Tables

**Table 1:** Patient demographics.

	Age (Years Old)	Height (cm) *	Weight (kg) *	BMI
Males	67.5 ± 9.4	165.9 ± 5.3	66.9 ± 12.9	24.2 ± 3.8
Females	64.0 ± 12.1	153.2 ± 6.5	58.4 ± 10.3	24.9 ± 3.9
Total	65.3 ± 11.3	157.6 ± 8.6	61.3 ± 11.9	24.6 ± 3.8

* *p* < 0.05 between the sexes.

**Table 2:** The results of EDX.

	Category	Symptomatic Side	Asymptomatic Side
Distal latency *	1	3	25
	2	26	42
	3	22	4
	4	7	0
	5	3	0
	6	15	0
Sensory nerve conduction velocity *	1	18	51
	2	21	11
	3	1	0
	4	27	1

* *p* < 0.05 between symptomatic and asymptomatic side.

## Data Availability

The datasets analyzed during the present study are available from the corresponding author upon reasonable request.
